# Pituitary adenylate cyclase-activating polypeptide (PACAP) inhibits the slow afterhyperpolarizing current sI_AHP_ in CA1 pyramidal neurons by activating multiple signaling pathways

**DOI:** 10.1002/hipo.22201

**Published:** 2013-09-30

**Authors:** Ruth DT Taylor, Marita Grønning Madsen, Michael Krause, Marisol Sampedro-Castañeda, Martin Stocker, Paola Pedarzani

**Affiliations:** 1 Research Department of Neuroscience, Physiology and Pharmacology, University College London,London, United Kingdom; 2 Department of Molecular Biology of Neuronal Signals, Max Planck Institute for Experimental Medicine,37075, Göttingen, Germany

**Keywords:** hippocampus, slow afterhyperpolarization, neuropeptide, protein kinase A, p38 MAP kinase

## Abstract

The slow afterhyperpolarizing current (sI_AHP_) is a calcium-dependent potassium current that underlies the late phase of spike frequency adaptation in hippocampal and neocortical neurons. sI_AHP_ is a well-known target of modulation by several neurotransmitters acting via the cyclic AMP (cAMP) and protein kinase A (PKA)-dependent pathway. The neuropeptide pituitary adenylate cyclase activating peptide (PACAP) and its receptors are present in the hippocampal formation. In this study we have investigated the effect of PACAP on the sI_AHP_ and the signal transduction pathway used to modulate intrinsic excitability of hippocampal pyramidal neurons. We show that PACAP inhibits the sI_AHP_, resulting in a decrease of spike frequency adaptation, in rat CA1 pyramidal cells. The suppression of sI_AHP_ by PACAP is mediated by PAC_1_ and VPAC_1_ receptors. Inhibition of PKA reduced the effect of PACAP on sI_AHP_, suggesting that PACAP exerts part of its inhibitory effect on sI_AHP_ by increasing cAMP and activating PKA. The suppression of sI_AHP_ by PACAP was also strongly hindered by the inhibition of p38 MAP kinase (p38 MAPK). Concomitant inhibition of PKA and p38 MAPK indicates that these two kinases act in a sequential manner in the same pathway leading to the suppression of sI_AHP_. Conversely, protein kinase C is not part of the signal transduction pathway used by PACAP to inhibit sI_AHP_ in CA1 neurons. Our results show that PACAP enhances the excitability of CA1 pyramidal neurons by inhibiting the sI_AHP_ through the activation of multiple signaling pathways, most prominently cAMP/PKA and p38 MAPK. Our findings disclose a novel modulatory action of p38 MAPK on intrinsic excitability and the sI_AHP_, underscoring the role of this current as a neuromodulatory hub regulated by multiple protein kinases in cortical neurons. © 2013 The Authors. Hippocampus Published by Wiley Periodicals, Inc.

## INTRODUCTION

Action potentials in hippocampal pyramidal neurons are followed by an afterhyperpolarization (AHP) that has three kinetically distinct phases: fast (fAHP), medium (mAHP), and slow AHP (sAHP) (reviewed in Sah and Faber, 2002; Stocker et al., 2004). The sAHP has a time-course of seconds (1–5 s), is evident after bursts of action potentials, and its amplitude is enhanced with increasing number of spikes in the burst (Alger and Nicoll, 1980; Hotson and Prince, 1980; Schwartzkroin and Stafstrom, 1980; Lancaster and Adams, 1986). The sAHP is mediated by a slow calcium-dependent potassium current, sI_AHP_, that contributes to the late phase of spike frequency adaptation in hippocampal pyramidal neurons (Madison and Nicoll, 1982; Lancaster and Adams, 1986; Lancaster and Nicoll, 1987). A trademark feature of the sI_AHP_ is its modulation by several neurotransmitters activating various signal transduction pathways. The modulation of sI_AHP_ by monoamine transmitters through the cyclic AMP (cAMP)-protein kinase A (PKA) pathway has been extensively studied (reviewed in Stocker et al., 2004). Beside monoamine transmitters, some neuropeptides expressed in interneurons and released in the hippocampal formation inhibit the sI_AHP_ and reduce spike frequency adaptation in pyramidal neurons. These include the vasoactive intestinal peptide (VIP, Haas and Gahwiler, 1992), the corticotropin releasing factor (CRF, Aldenhoff et al., 1983) and the calcitonin gene-related peptide (CGRP, Haug and Storm, 2000), all exerting their inhibitory action on sI_AHP_ via the cAMP-PKA pathway (Haug and Storm, 2000).

The neuropeptide pituitary adenylate cyclase-activating polypeptide (PACAP) belongs to a superfamily of structurally related peptide hormones that include glucagon, glucagon-like peptides, secretin and growth hormone-releasing hormone (Harmar, 2001). PACAP consists of two isoforms, PACAP-27 with 27 and PACAP-38 with 38 amino acids, sharing identical sequences for the initial 27 amino acids. Both isoforms were first identified and isolated from ovine hypothalamic tissue (Miyata et al., 1989,1990) and subsequently found to be preserved from protochordates to mammals (Vaudry et al., 2009). PACAP exhibits a wide range of functions and acts as a neurotransmitter and trophic factor in the CNS (Vaudry et al., 2009). The first 28 amino acids of PACAP-38 show 68% sequence identity with VIP (Miyata et al., 1989,1990), suggesting that VIP and PACAP might mediate their biological effects through shared receptors. Two classes of PACAP receptors have been characterized based on their relative affinity for PACAP and VIP. PACAP has a potency >100-fold higher than VIP at the recombinant PAC_1_ receptor, whereas it displays a potency similar to VIP at the recombinant VPAC receptors (VPAC_1_ and VPAC_2_) in radioligand binding assays (reviewed in Vaudry et al., 2009; Harmar et al., 2012). PAC_1_ and VPAC receptors are coupled to the heterotrimeric G-protein Gα_s_ and increase cAMP levels (reviewed in Harmar, 2001).

PACAP and its receptors are widely expressed in the CNS. PACAP mRNA (Skoglosa et al., 1999; Jaworski and Proctor, 2000; Hannibal, 2002) and immunoreactivity (Koves et al., 1991; Piggins et al., 1996; Hannibal, 2002) have been observed in the rat hippocampus, in the cell body layers of the CA1-CA3 fields, in dentate gyrus granule cells, mossy cells and pyramidal basket cells. Additionally, PACAP immunoreactive fibers have been detected both in the CA1-CA3 fields and in the dentate gyrus. In the hippocampus, PACAP might therefore be released by neurons (pyramidal cells or interneurons) within the hippocampal formation and/or by afferents coming from PACAP-rich regions, such as the hypothalamus (Piggins et al., 1996). Northern blot analysis and *in situ* hybridization revealed the presence of PAC_1_ (Hashimoto et al., 1993; Spengler et al., 1993), VPAC_1_ (Ishihara et al., 1992) and VPAC_2_ (Lutz et al., 1993) receptor mRNA in the hippocampal formation. Moreover, PACAP binding sites and receptors were detected in the CA1-CA3 regions and dentate gyrus of the hippocampus in autoradiographic studies (Cauvin et al., 1991; Masuo et al., 1992). Immunohistochemistry showed that VPAC_2_ receptors were highly expressed in dentate gyrus granule cells and in CA1-CA3 pyramidal cells, whilst PAC_1_ and VPAC_1_ were expressed in the same cells but at lower levels (Joo et al., 2004).

The presence of PACAP and PACAP receptors in the hippocampus prompted us to hypothesize that PACAP might modulate the sI_AHP_ in hippocampal pyramidal neurons, thereby affecting their excitability and firing pattern. In this study we have investigated the effect of PACAP on the sI_AHP_ and the signal transduction pathway used in rat CA1 pyramidal neurons.

## MATERIALS AND METHODS

Male Sprague Dawley rats, 21-to 24-days old, were used to prepare transverse hippocampal brain slices. Rats were anesthetized with isoflurane and decapitated according to the UK Home Office Animal Scientific Procedures Act (1986), and protocols were reviewed and approved by the University College London Animal Ethical Committee.

Whole-cell patch clamp recordings were performed from CA1 pyramidal neurons using the blind patching technique (Blanton et al., 1989). All whole-cell patch clamp recordings were conducted with an EPC10 amplifier (HEKA, Germany) and the software Pulse for data acquisition. Slices were perfused with ACSF (in mM: 125 NaCl, 1.25 KCl, 2.5 CaCl_2_, 1.5 MgCl_2_, 1.25 KH_2_PO_4_, 25 NaHCO_3_, and 16 glucose) and the sI_AHP_ was recorded at room temperature with patch pipettes made of borosilicate glass and containing a gluconate-based intracellular solution (in mM: 135 K-gluconate, 10 KCl, 10 HEPES, 1 MgCl_2_, 2 ATP-Na, and 0.4 GTP-Na). The liquid junction potential for the intracellular solution is −11 mV. Voltage values reported here are not corrected for the liquid junction potential. Only cells with a resting membrane potential ≤−50 mV and a series resistance ≤30 MΩ were included in this study.

The sI_AHP_ was elicited by stepping to +10–30 mV for 100–150 ms from a holding potential of −50 mV. Recordings were performed in the presence of 0.5 µM tetrodotoxin (TTX) to block voltage-gated sodium channels, and 1 mM tetraethylammonium (TEA) to block a subset of voltage-gated potassium channels to increase the calcium influx and thereby the calcium dependent sI_AHP_. Traces were filtered at 0.25 kHz and sampled at 1.25 kHz and the stimulus was repeated with a 30 s interval. Bovine serum albumin (BSA) was added to the ACSF (25 µg ml^−1^) when peptides were used extracellularly to minimize unspecific binding. Peptides were applied by bath perfusion. It is worth noticing that the actual concentration of the peptide/s at the relevant receptors might differ from that nominally applied, due to the size and diffusion of the peptide/s to neurons located at different depths in the brain slice and, possibly, to the action of extracellular peptidases. Protein kinase inhibitors were applied intracellularly through the patch pipette. DMSO up to 0.13% did not affect either the sI_AHP_ amplitude or charge transfer, or the effect of PACAP-27 on the sI_AHP_ amplitude or charge transfer when applied intracellularly through the patch pipette as a vehicle to dissolve some protein kinase inhibitors.

Cells were excluded if the series resistance changed significantly (>25%) in the course of experiments. Modulation of sI_AHP_ was determined as changes in amplitude and charge transfer of sI_AHP_. The sI_AHP_ amplitude was measured by calculating the mean current between two cursors placed 50 ms apart, 1 s after the initiation of the voltage step to avoid contamination by other, faster potassium currents. Charge transfer was determined as the area under the curve starting from the sI_AHP_ peak until full decay had occurred.

Isoproterenol hydrochloride and tetraethylammonium chloride (TEA) were obtained from Sigma (UK); PACAP-27 amide, SB 203580, phorbol-12,13-dibutyrate (PDBu), bisindolylmaleimide I (BIM-1), and chelerythrine chloride from Calbiochem (now Merck Serono Ltd, Middlesex, UK); PACAP-38 from Peninsula Laboratories Europe (St. Helens, UK); PACAP-(6–38) from Bachem (Bubendorf, Switzerland); tetrodotoxin (TTX) from Latoxan (Valence, France); and Rp-cAMPS and 8-(4-chloro-phenylthio)−2'-O-methyl-cAMP (8CPT-O-Me-cAMP) from BioLog Life Science Institute (Bremen, Germany). Maxadilan and max.d.4 were a generous gift from Dr. Ethan Lerner at Harvard Medical School.

Statistical analysis was performed with GraphPad InStat. Data are presented as mean with error bars indicating the standard error of the mean (S.E.M.), when not otherwise specified.

## RESULTS

The sI_AHP_ was recorded in the whole-cell patch-clamp configuration from 152 CA1 pyramidal neurons. CA1 neurons had an average resting membrane potential of −59.8 ± 3.5 mV (*n* = 84; mean ± SD) and an input resistance of 175.4 ± 51.1 MΩ (*n* = 80; mean ± SD). The sI_AHP_ amplitude was on average 40.6 ± 24.2 pA and the charge transfer 101.4 ± 63.8 pC (*n* = 84; mean ± SD).

### PACAP Suppresses sI_AHP_ in CA1 Pyramidal Neurons

**Figure 1:**
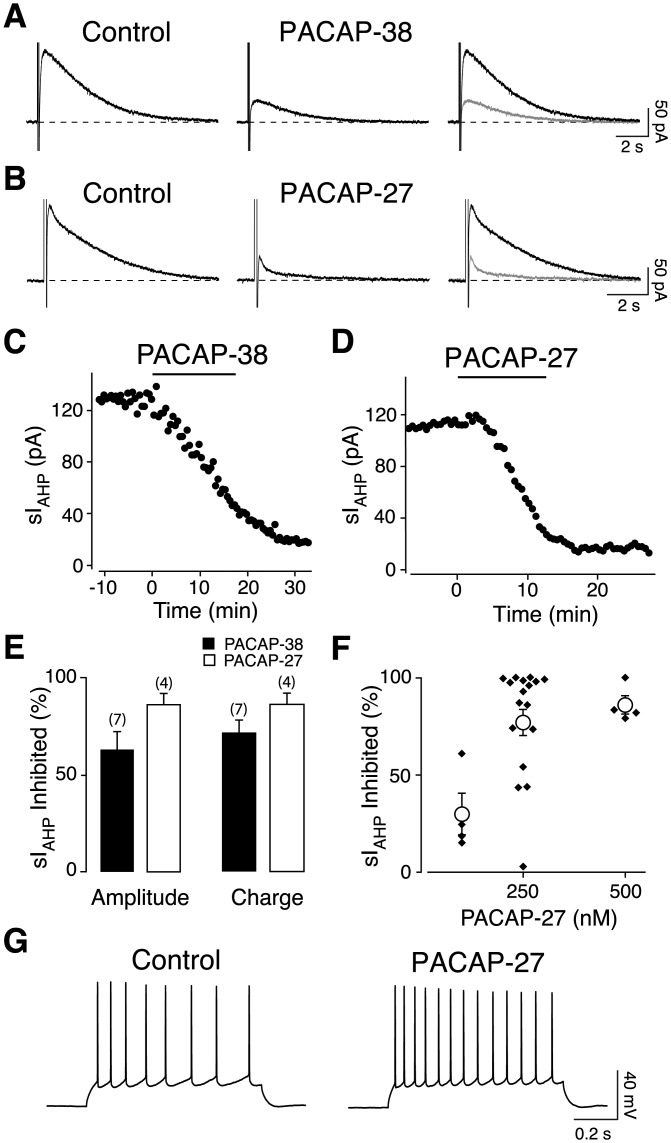
PACAP-38 and PACAP-27 suppress sI_AHP_ in hippocampal neurons. Averaged current traces (*n* = 3) of the sI_AHP_ before (control) and after application of 500 nM PACAP-38 (A) or 500 nM PACAP-27 (B). Overlaid traces are shown in the rightmost panels (A and B), control (black) and PACAP (grey). The dashed line corresponds to the baseline current. Time course of the effect of PACAP-38 (C) and PACAP-27 (D) on the sI_AHP_ amplitude from the experiments shown in A and B. Bars indicate the presence of 500 nM PACAP in the bath solution. (E) Effect of 500 nM PACAP-38 (black) and PACAP-27 (white) on sI_AHP_ amplitude and charge transfer. No significant difference was observed between the two PACAP isoforms. (F) The effect of PACAP-27 on sI_AHP_ was concentration-dependent. PACAP-27 significantly suppressed sI_AHP_ amplitude at both 250 nM (*n* = 18, *P* < 0.0001) and 500 nM (*n* = 4, *P* < 0.01), while at 100 nM PACAP-27 did not cause a significant inhibition of sI_AHP_ (*n* = 4, *P* > 0.05). The difference between the sI_AHP_ reduction caused by 250 and 500 nM PACAP-27 was not significant (*P* > 0.05, Dunn's multiple comparisons test). (G) PACAP-27 (250 nM) decreased spike frequency adaptation in a representative CA1 neuron in response to a current injection of 140 pA, resting membrane potential = −59 mV. Similar effects were observed in seven cells.

The modulatory effects on the sI_AHP_ of VIP and other neuropeptides that activate the cAMP-PKA pathway in hippocampal pyramidal neurons are well studied (Aldenhoff et al., 1983; Haas and Gahwiler, 1992; Haug and Storm, 2000). Given the structural similarity of PACAP and VIP (Miyata et al., 1989; Miyata et al., 1990) and the fact that they share a subset of receptors (VPAC_1_ and VPAC_2_), coupled to the cAMP-PKA pathway (reviewed in Vaudry et al., 2009), we hypothesized that PACAP might suppress sI_AHP_ in hippocampal pyramidal neurons. We found that PACAP-38 (500 nM) reduced the sI_AHP_ amplitude (Figs. 1A,C) by 67% ± 5.9% (Fig. 1E, *n* = 7) and the charge transfer by 77.3% ± 5.2% (Fig. 1E, *n* = 7) in CA1 pyramidal neurons.

The region responsible for receptor activation of PACAP-38 resides in its N-terminal 27-amino acid sequence, which corresponds to the naturally occurring isoform PACAP-27 (Miyata et al., 1990). We therefore tested the effect of PACAP-27 (500 nM) on sI_AHP_. PACAP-27 suppressed sI_AHP_ within 15–20 min of bath application (Fig. 1B,D), and no reversibility was observed within 15–35 min of wash-out. PACAP-27 suppressed the sI_AHP_ amplitude by 86.1% ± 4.7% and the charge transfer by 85.8% ± 5.1% (Fig. 1E, *n* = 4). No significant difference between PACAP-27 and PACAP-38 was found with regard to the reduction in sI_AHP_ amplitude (*P* > 0.06, unpaired *t* test) and charge transfer (*P* > 0.3, unpaired *t* test). We therefore decided to use PACAP-27 in the following experiments.

PACAP-27 suppressed the sI_AHP_ in a concentration dependent manner (Fig. 1F). While 100 nM PACAP-27 only slightly reduced the sI_AHP_ amplitude (29.9% ± 10.6%, *n* = 4), the reduction with 250 nM was more substantial (77.4% ± 6.7%, *n* = 18) and comparable to the effect of 500 nM PACAP-27 (86.1% ± 4.7%, *n* = 4). PACAP-27 at 250 nM caused a suppression of the sI_AHP_ amplitude by more than 90% in 50% of the neurons (9 out of 18 cells; Fig. 1F). 2 out of 18 cells displayed a sI_AHP_ inhibition comprised between 85 and 90%; 2 out of 18 cells between 70 and 75%; 4 out of 18 cells between 40 and 55% (Fig. 1F). In one case, corresponding to 5.6% of all cells tested, the sI_AHP_ was not affected by 250 nM PACAP-27 (2.4% inhibition; Fig. 1F). All cells treated with 500 nM PACAP-27 displayed reductions of the sI_AHP_ amplitude in the range of 80–100% (*n* = 4; Fig. 1F).

Beside inhibiting sI_AHP_, PACAP produced an inward shift in the holding current at the holding potential of −50 mV, corresponding to a depolarizing effect. PACAP-38 at 500 nM elicited an inward current of 16.0 ± 2.1 pA in 7 out of 7 cells (*n* = 7; *P* = 0.0002). An inward current of 14 ± 3.3 pA (*n* = 18; *P* = 0.0006) was observed in response to 250 nM PACAP-27 in 16 out of 18 cells. The inward current might be due to the modulatory action of PACAP on background (leak) channels, HCN channels, and/or other nonspecific cationic channels, in analogy to what has been observed for other neurotransmitters inhibiting the sI_AHP_ in hippocampal neurons.

PACAP-27 at 250 nM did not significantly affect the input resistance of CA1 pyramidal neurons, which was 164.5 ± 9.7 MΩ before and 163.9 ± 8.1 MΩ after PACAP-27 application (*n* = 18; *P* = 0.96, Wilcoxon matched-pairs signed-ranks test).

The inhibition of the sI_AHP_ reduces late spike frequency adaptation in hippocampal pyramidal neurons (Madison and Nicoll, 1982; Madison and Nicoll, 1984). Application of 250 nM PACAP-27 reduced spike frequency adaptation in CA1 pyramidal neurons (Fig. 1G, *n* = 7), in agreement with its inhibitory effect on the sI_AHP_.

### Activation of PAC-1 Receptors Partially Mimics the Effect of PACAP on sI_AHP_

**Figure 2:**
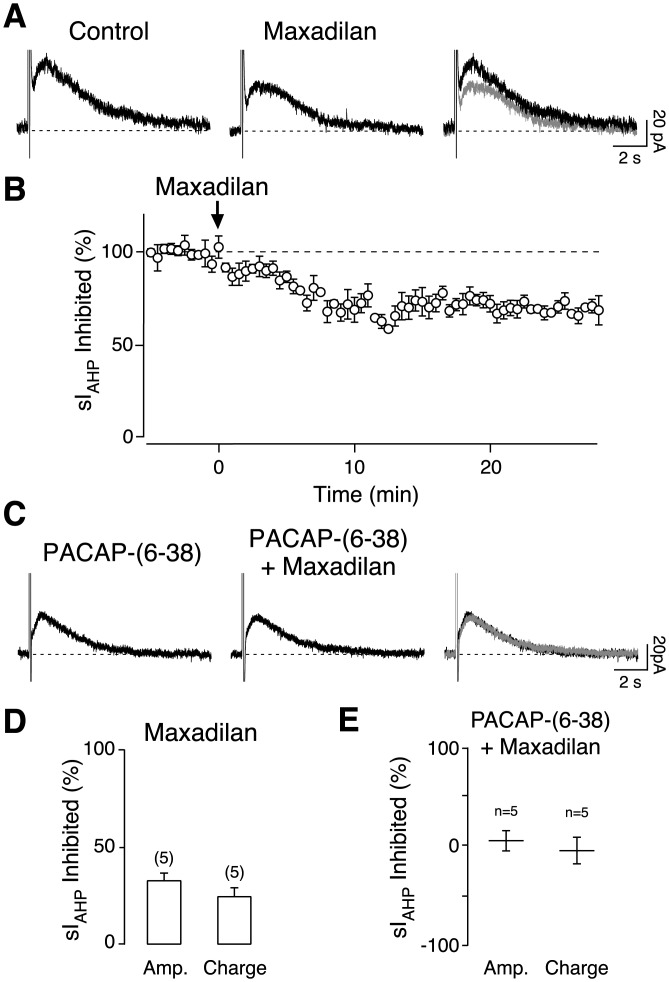
Selective activation of PAC_1_ receptors partly mimics the inhibitory effect of PACAP on the sI_AHP_. Averaged current traces (*n* = 3) of the sI_AHP_ before (control) and after application of the selective PAC_1_ receptor agonist maxadilan at 250 nM (A). In the rightmost panel, the traces in the absence (black) and presence (grey) of maxadilan are superimposed. The dashed line corresponds to the baseline current. (B) Time-course of the effect of 250 nM maxadilan on the amplitude of sI_AHP_. Each point is the mean ± SEM of five experiments. Application of maxadilan is indicated by the arrow. (C) 500 nM PACAP-(6–38), a PAC_1_ receptor antagonist, prevented the effect of 250 nM maxadilan as shown on averaged current traces (*n* = 3) of the sI_AHP_. The dashed line corresponds to the baseline current. Overlaid traces are shown in the rightmost panel. Bar charts summarizing the reduction of sI_AHP_ amplitude and charge transfer by 250 nM maxadilan (D) and the absence of maxadilan effect in the presence of 500 nM PACAP-(6–38) (E) in five cells. Error bars indicate S.E.M.

Neuronal PACAP receptors comprise PAC_1_, selective for PACAP and coupled to both adenylyl cyclases and phospholipase C, and VPAC_1_ and VPAC_2_ receptors, activated by both PACAP and VIP and coupled to adenylyl cyclases (Vaudry et al., 2009). All three PACAP receptor subtypes are expressed in CA1 pyramidal cells (Hashimoto et al., 1996; Shioda et al., 1997; Joo et al., 2004). To elucidate the specific contribution of PAC_1_ receptors to the PACAP-mediated sI_AHP_ inhibition in CA1 pyramidal cells, we used the PAC_1_ selective agonist maxadilan, a peptide structurally unrelated to PACAP (Lerner et al., 1991), which has no activity at VPAC_1_ and VPAC_2_ receptors (Moro and Lerner, 1997). Maxadilan and PACAP activate PAC_1_ receptors with a similar potency in the rat brain (Moro et al., 1996). When tested at 250 nM, the same concentration as used for PACAP-27, maxadilan reduced the sI_AHP_ amplitude (Figs 2A,B) by 32.6% ± 3.9% (*n* = 5; *P* = 0.001, *t* test) and charge transfer by 24.4% ± 4.7% (*n* = 5; *P* = 0.007, *t* test) (Fig. 2D). This indicates that PAC_1_ receptors contribute to the PACAP-induced suppression of sI_AHP_ in CA1 pyramidal neurons.

To investigate whether the reduction of sI_AHP_ induced by maxadilan could be prevented by a PACAP receptor antagonist, we used PACAP-(6–38), a truncated PACAP-38 lacking six amino terminal amino acids. PACAP-(6–38) is a selective antagonist at PAC_1_ and VPAC_2_ receptors (Dickinson et al., 1997; Moro et al., 1999). In the presence of PACAP-(6–38) (500 nM), application of maxadilan (250 nM) did not significantly reduce the sI_AHP_ amplitude (5.7% ± 10.7%, *n* = 4, *P* > 0.5, *t* test) or charge transfer (−5.3% ± 13.6%, *n* = 4, *P* > 0.5 *t* test) (Fig. 2C,E). The lack of reduction of the sI_AHP_ by a specific PAC_1_ receptor agonist (maxadilan) in the presence of a PAC_1_ receptor antagonist further supports a contribution by PAC_1_ receptors.

Surprisingly, the reduction of the sI_AHP_ as a consequence of PAC_1_ receptor activation is not supported by the results obtained upon 250 nM PACAP-27 application in the presence of PACAP-(6–38). Under this condition, PACAP-27 reduced the sI_AHP_ amplitude (79.2% ± 11.2%, *n* = 5, *P* = 0.002, *t* test) and the charge transfer (86.8% ± 8.5%, *n* = 5, *P* = 0.0005, *t* test) to a comparable extent (amplitude: *P* > 0.5, charge transfer: *P* > 0.3) as observed in the absence of PACAP-(6–38) (Fig. 1). The suppression of sI_AHP_ by maxadilan but the lack of antagonism of PACAP-27 by PACAP-(6–38) suggests the activation of more than one receptor subtype in the PACAP-mediated inhibition of this current.

Max.d.4 is a derivative of maxadilan, in which nineteen amino acids between positions 24 and 42 are deleted. It is a selective PAC_1_ receptor antagonist that is more potent than PACAP-(6–38) (Moro et al., 1999). In the presence of 500 nM max.d.4, 250 nM PACAP-27 inhibited the sI_AHP_ amplitude (69.8% ± 10.8%, *n* = 4, *P* < 0.01, *t* test) and charge transfer (74.5% ± 7.3%, *n* = 4, *P* < 0.01, *t* test) to an extent that was comparable (amplitude and charge transfer: *P* > 0.5) to the effect of PACAP-27 alone (Fig. 1). This further indicates that antagonism of PAC_1_ receptors alone is not sufficient to prevent the PACAP-27 mediated inhibition of sI_AHP_, which is instead due to the activation of more than just PAC_1_ receptors.

### PACAP-27 Suppresses sI_AHP_ Partly Through PKA Activation

In hippocampal neurons monoaminergic neurotransmitters such as noradrenaline, histamine, serotonin and dopamine inhibit the sI_AHP_ through PKA activation (Pedarzani and Storm, 1993,1995). Moreover, in CA1 pyramidal neurons CRF, VIP and CGRP have been shown to suppress sI_AHP_ in a PKA-dependent manner (Haug and Storm, 2000). We therefore asked whether PKA was required to mediate the inhibitory effect of PACAP on sI_AHP_ in hippocampal pyramidal neurons.

**Figure 3:**
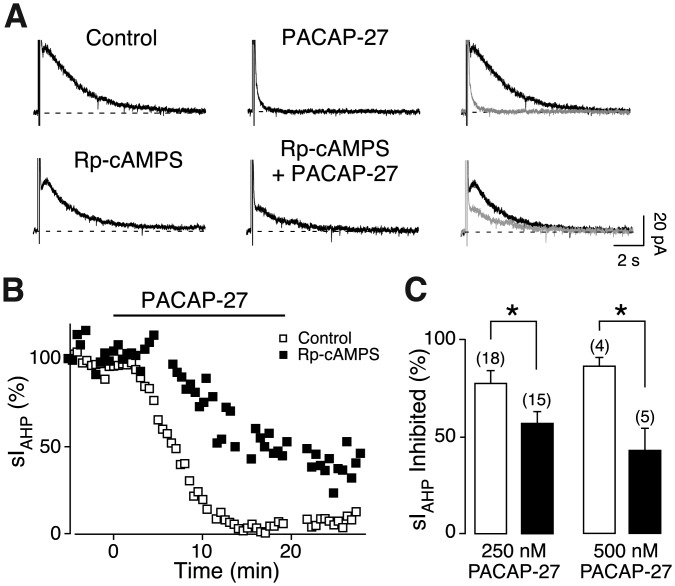
PKA activation contributes to the inhibition sI_AHP_ by PACAP-27. Averaged current traces (*n* = 3) of the sI_AHP_ before and after bath application of 250 nM PACAP-27 in the absence (upper traces) and in the presence (lower traces) of the PKA inhibitor Rp-cAMPS (500 µM) (A). In the rightmost panels, the traces in the absence (black) and presence (grey) of PACAP-27 are superimposed. The dashed line corresponds to the baseline current. (B) Time course of the normalized sI_AHP_ amplitude from the experiments shown in A before, during, and after PACAP-27 application in the presence (black squares) and absence (white squares) of intracellular Rp-cAMPS. Bar indicates the presence of 250 nM PACAP-27. (C) Bar chart summarizing the effect on the sI_AHP_ amplitude of PACAP-27 at 250 and 500 nM in the absence (white bars) and presence (black bars) of Rp-cAMPS. Rp-cAMPS significantly reduced the suppression of sI_AHP_ amplitude by PACAP-27 at 250 nM (*P* = 0.04, *n* = 15, Mann–Whitney test), and at 500 nM (*P* = 0.01, *n* = 5, unpaired *t* test). The values for sI_AHP_ inhibition by PACAP-27 at 250 nM (*n* = 18) and 500 nM (*n* = 4) in the absence of Rp-cAMPS are the same as in Figure 1E,F and are reported here for comparison. Error bars indicate S.E.M. * indicates statistical significance.

To address this question, we used the PKA inhibitor Rp-cAMPS added to the intracellular solution (Pedarzani and Storm, 1993). Application of PACAP-27 (250 nM) in the presence of Rp-cAMPS (500 µM) caused an inhibition of the sI_AHP_ that was attenuated when compared to the effect of PACAP-27 alone (Fig. 3A,B). The inhibition of the sI_AHP_ amplitude was reduced from 77.4% ± 6.7% in the absence of Rp-cAMPS (Figs. 1F and 3C) to 56.8% ± 5.9% in the presence of Rp-cAMPS (Fig. 3C). Similarly, the sI_AHP_ inhibition by 500 nM PACAP-27 was reduced from 86.1% ± 4.7% in the absence (Fig. 1F and Fig. 3C) to 42.7% ± 11.1% in the presence of Rp-cAMPS (Fig. 3C). An inward shift in the holding current of 16.6 ± 6.8 pA (*n* = 15; *P* = 0.002) was observed upon application of PACAP-27 (250 nM) in the presence of Rp-cAMPS.

To ensure that Rp-cAMPS inhibited PKA under our experimental conditions, we used isoproterenol (1 µM), a β-adrenergic receptor agonist that inhibits the sI_AHP_ by activation of the cAMP/PKA pathway (Pedarzani and Storm, 1993). The sI_AHP_ inhibiton caused by isoproterenol (100% ± 0.02%, *n* = 4) was significantly reduced in the presence of Rp-cAMPS (41.2% ± 8.4%, *n* = 5, *P* = 0.0005, unpaired *t* test). When isoproterenol was applied after PACAP-27 in the presence of Rp-cAMPS, the sI_AHP_ was inhibited to 43.1% ± 7% (*n* = 5) of the amplitude left after PACAP-27 inhibition, showing that Rp-cAMPS inhibited PKA. These results show that PKA contributes to the PACAP-27-mediated inhibition of the sI_AHP_.

### Are MAP Kinases Involved in the PACAP-27-Mediated Inhibition of sI_AHP_?

In a Drosophila mutant PACAP modulates a potassium current through the activation of a mitogen-activated protein (MAP) kinase pathway (Zhong, 1995). PACAP has also been shown to activate the MAP kinase kinase (MEK)/extracellular-signal-regulated kinase (ERK) pathway in cultured cerebellar granule cells (Villalba et al., 1997). Furthermore, PACAP-38 activates p38 MAP kinase (p38 MAPK) in mouse cultured cerebellar granule cells (Ster et al., 2007). We therefore asked whether the effect of PACAP on the sI_AHP_ could be mediated by the activation of the ERK or p38 MAPK pathway.

To first test for a potential contribution of the MEK-ERK1/2 pathway, we applied intracellularly the MEK inhibitor UO126 (40 µM) (Favata et al., 1998). In the presence of UO126, PACAP-27 (250 nM) inhibited the sI_AHP_ (58.5% ± 8.7%, *n* = 8) to the same extent as in intercalated control experiments performed with 0.13% DMSO in the pipette solution (63.1% ± 7.1%, *n* = 6, *P* > 0.6, Mann–Whitney test). Similarly, inhibition of MEK by UO126 did not affect the suppression of sI_AHP_ by 500 nM PACAP-27 (90.2% ± 4.4%, *n* = 6, *P* > 0.1, Mann–Whitney test).

**Figure 4:**
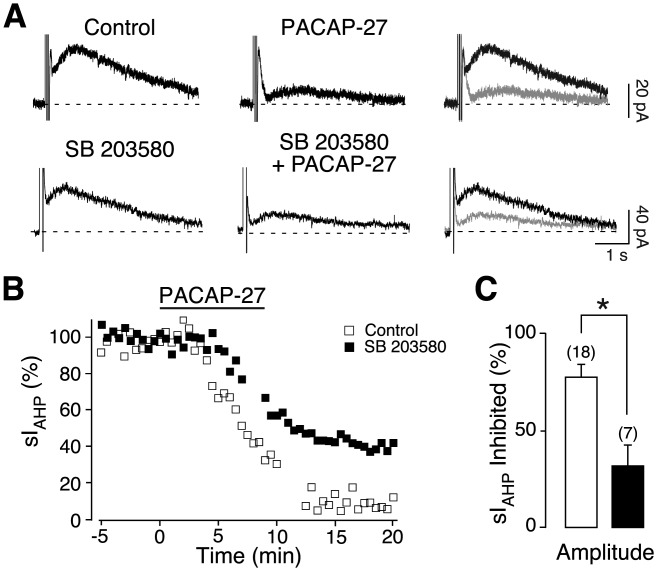
p38 MAPK activation is involved in the PACAP-27-induced inhibition of sI_AHP_. Averaged current traces (*n* = 3) of the sI_AHP_ before and after application of 250 nM PACAP-27 in the absence (upper traces) and in the presence (lower traces) of the p38 MAPK inhibitor SB 203580 (20 µM) in the intracellular solution (A). The rightmost panels show superimposed traces in the absence (black) and presence (grey) of PACAP-27. The dashed line corresponds to the baseline current. (B) Time course of the effect of PACAP-27 on the normalized sI_AHP_ amplitude from the experiments in A, in the absence (white squares) and in the presence (black squares) of SB 203580. Bar indicates the presence of 250 nM PACAP-27. (C) Bar chart summarizing the relative inhibition of the sI_AHP_ amplitude by 250 nM PACAP-27 under control conditions (*n* = 18; white bar) and in the presence of SB 203580 (*n* = 7; black bar). SB 203580 markedly inhibited the suppression of sI_AHP_ mediated by PACAP-27. Error bars indicate S.E.M. * indicates statistical significance.

Next, we tested the hypothesis of an involvement of p38 MAPK in the PACAP-mediated inhibition of sI_AHP_. In the presence of SB 203580 (20 µM), a p38 MAPK inhibitor (Eyers et al., 1998), PACAP-27 (250 nM) partly inhibited the sI_AHP_ (Fig. 4A). Figure 4B shows the time course of the reduction in sI_AHP_ amplitude from the experiment in Figure 4A before, during, and after PACAP-27 application. When compared with experiments performed in the absence of SB 203580, the inhibition of the sI_AHP_ amplitude by PACAP-27 was reduced in the presence of the p38 MAPK inhibitor (31.3% ± 11.2%, *n* = 7, *P* = 0.006, Mann–Whitney test, Fig. 4C). Similar experiments were performed with an increased concentration of PACAP-27 (500 nM). In this case SB 203580 did not reduce the PACAP-27-induced inhibition of the sI_AHP_ amplitude (70.4% ± 11.6%) when compared with the sI_AHP_ inhibition induced in the absence of the p38 MAPK inhibitor (*n* = 5, *P* > 0.2, unpaired *t* test). SB 203580 largely prevented the inward shift in the holding current (4.8 ± 2.9 pA; *n* = 7; *P* = 0.14) caused by PACAP-27 (250 nM).

Taken together, these results indicate that p38 MAPK is involved in mediating the inhibitory effect of PACAP on the sI_AHP_ in CA1 pyramidal neurons, while a contribution by the MEK/ERK1/2 pathway seems unlikely.

### Does PACAP Activate p38 MAP Kinase and PKA in the Same or in Parallel Pathways Resulting in sI_AHP_ Inhibition?

Both the inhibition of PKA by Rp-cAMPS (Fig. 3) and of p38 MAPK by SB 203580 (Fig. 4) result in a decrease of the PACAP-27-induced sI_AHP_ suppression. This prompts the question as to whether PACAP-27 activates p38 MAPK and PKA as part of the same or two parallel pathways leading to sI_AHP_ suppression. If PKA and p38 MAPK operate sequentially as part of the same pathway, their concomitant inhibition should not have a stronger impact on the effect of PACAP-27 than inhibition of each single kinase. Conversely, if PKA and p38 MAPK act through parallel, independent pathways their combined inhibition should have a stronger impact on the effect of PACAP-27.

**Figure 5:**
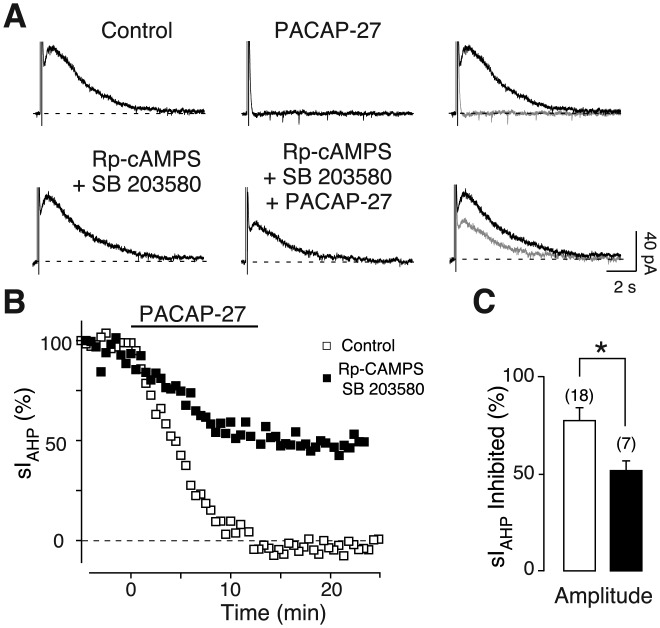
Simultaneous inhibition of p38 MAPK and PKA partly prevents the suppression of sI_AHP_ mediated by PACAP-27. Averaged current traces (*n* = 3) of the sI_AHP_ in the absence and presence of 250 nM PACAP-27 recorded without (upper traces) and with (lower traces) the PKA inhibitor Rp-cAMPS (500 µM) and the p38 MAP kinase inhibitor SB 203580 (20 µM) in the patch pipette (A). The rightmost panels display the superimposed traces in the absence (black) and presence (grey) of PACAP-27. The dashed line corresponds to the baseline current. (B) Time course of the effect of PACAP-27 on the sI_AHP_ amplitude from the experiments in A, with (black squares) and without (white squares) Rp-cAMPS + SB 203580. Bar indicates the application of 250 nM PACAP-27. (C) Bar chart summarizing the relative inhibition of sI_AHP_ by 250 nM PACAP-27 under control conditions (*n* = 18; white bar) and in the presence of Rp-cAMPS + SB 203580 (*n* = 7; black bar). Coapplication of Rp-cAMPS and SB 203580 partly prevented the suppression of sI_AHP_ amplitude mediated by 250 nM PACAP-27. Error bars indicate S.E.M. * indicates statistical significance.

The PKA inhibitor Rp-cAMPS and the p38 MAPK inhibitor SB 203580 were included together in the intracellular solution, and 250 nM PACAP-27 was applied for ∼15 min, causing a partial inhibition of the sI_AHP_ (Fig. 5B). The PACAP-27-induced inhibition of sI_AHP_ was attenuated in the presence of Rp-cAMPS and SB 203580 (Fig. 5A). The reduction of the PACAP-27 effect on the sI_AHP_ upon combined inhibition of PKA and p38 MAPK was significant compared with control conditions (51.8% ± 4.9%; *n* = 7; *P* = 0.02, Mann–Whitney test; Fig. 5C). However, the PACAP-27-mediated suppression of sI_AHP_ amplitude observed in the presence of Rp-cAMPS and SB 203580 was not significantly different from that observed in the presence of Rp-cAMPS alone (compare Figs. 5C and 3C) or SB 203580 alone (compare Figs. 5C and 4C) (Kruskal–Wallis Test—nonparametric ANOVA: *P* = 0.105). The observation that the combined inhibition of PKA and p38 MAPK does not prevent the PACAP-27 reduction of the sI_AHP_ to a larger extent than the inhibition of each single kinase suggests that they act sequentially as part of the same signal transduction pathway.

**Figure 6:**
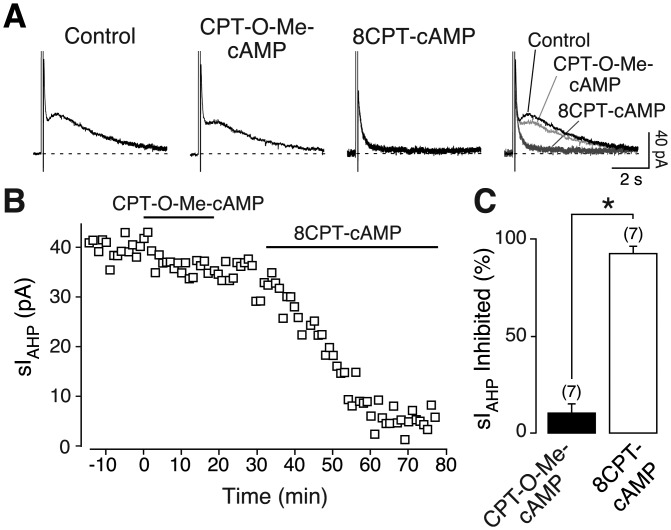
Activation of EPACs does not suppress sI_AHP_ in CA1 neurons. Averaged sI_AHP_ traces (*n* = 3) recorded in the absence (control) and presence of the EPAC superactivator 8CPT-O-Me-cAMP (5 µM) and of the PKA activator 8CPT-cAMP (5 µM) (A). The rightmost panel shows the same traces superimposed. (B) Time course of action of 8CPT-O-Me-cAMP (5 µM) and 8CPT-cAMP (5 µM) on the sI_AHP_ amplitude in the same cell as in A. (C) Bar diagram summarizing the effects of 8CPT-O-Me-cAMP (5 µM; black bar) and subsequently applied 8CPT-cAMP (5–50 µM; white bar) on the sI_AHP_ amplitude in seven cells. The 8CPT-cAMP was used at a concentration of 50 µM in 6 out of 7 cells. The suppression of sI_AHP_ caused by 8CPT-cAMP was significantly larger than that observed in response to 8CPT-O-Me-cAMP (*P* < 0.0001, *n* = 7, unpaired *t* test). Error bars indicate S.E.M. * indicates statistical significance.

cAMP activates not only PKA but also “exchange proteins directly activated by cAMP” (EPAC) (Rehmann et al., 2003), which can lead to the activation of small G-proteins and p38 MAPK (Shi et al., 2006; Ster et al., 2007). Activation of p38 MAPK by EPACs would be in parallel to the one by PKA (Fig. 8). If indeed PKA and p38 MAPK act sequentially in the same pathway to mediate the inhibition of sI_AHP_ by PACAP-27, as suggested by the combined inhibition of PKA and p38 MAPK (Fig. 5), we would expect the EPAC contribution to this pathway to be minor or negligible. To distinguish between PKA and EPAC-mediated effects, we applied the highly specific EPAC superactivator 8CPT-O-Me-cAMP (Rehmann et al., 2003). 8CPT-O-Me-cAMP (5 µM) reduced the sI_AHP_ amplitude by 10.3% ± 5.2% (Fig. 6A–C, *n* = 7). This reduction was not significant (*P* > 0.05). To assess the integrity of the PKA meditated modulation of the sI_AHP_ we applied to the same cells the membrane permeable PKA activator 8CPT-cAMP (Fig. 6A,B). PKA activation by 8CPT-cAMP led to a suppression of the sI_AHP_ by 92.8% ± 3.4% (*n* = 7, Fig. 6C), showing the functionality of the PKA pathway. The lack of current inhibition by EPAC activation excludes a contribution by EPACs to the modulation of the sI_AHP_. This supports the finding that PKA and p38 MAPK are arranged sequentially to mediate the inhibition of the sI_AHP_ by PACAP (Fig. 8).

### PKC is not Involved in the sI_AHP_ Inhibition Mediated by PACAP-27

Besides their coupling to the cAMP/PKA and MAP kinase pathways, PACAP receptors have been shown to activate phospholipase C (PLC) linked signaling cascades (Spengler et al., 1993; Harmar, 2001; reviewed in Vaudry et al., 2009). Protein kinase C (PKC) is generally thought to act downstream of PLC (Nishizuka, 1992) and its activation suppresses the sI_AHP_ in hippocampal pyramidal neurons (Malenka et al., 1986). We therefore investigated whether, beside PKA and p38 MAPK, also PKC is involved in the sI_AHP_ suppression induced by PACAP-27.

**Figure 7:**
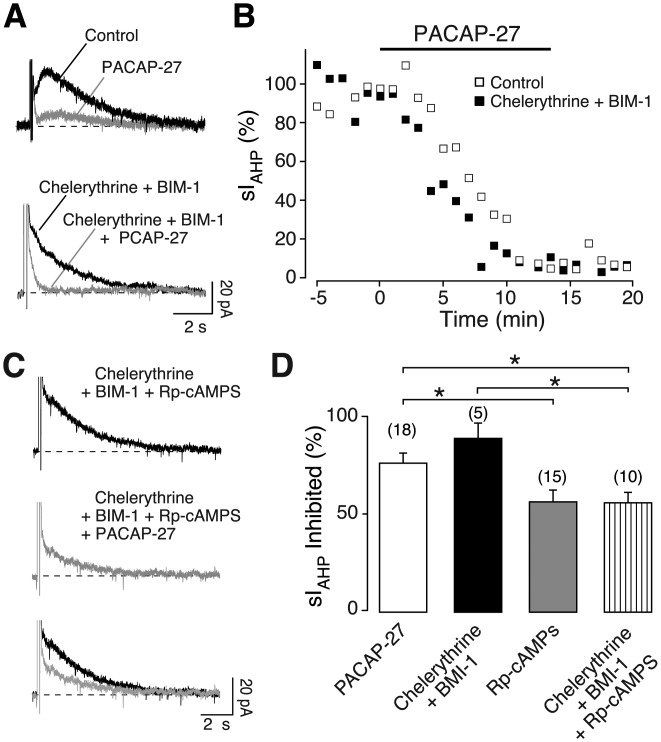
PKC is not involved in the sI_AHP_ suppression mediated by PACAP-27. Superimposed averaged sI_AHP_ traces (*n* = 3) recorded in the absence and presence of 250 nM PACAP-27 without (top panel) and with (lower panel) the PKC inhibitors chelerythrine (20 µM) and BIM-1 (500 nM) in the patch pipette (A). The dashed line corresponds to the baseline current. (B) Time course of the effect of PACAP-27 on the normalized sI_AHP_ amplitude from the experiments in A, with white squares indicating the absence and black squares the presence of Chelerythrine + BIM-1. Bar indicates the application of 250 nM PACAP-27. (C) Averaged sI_AHP_ traces (*n* = 3) recorded in the absence and presence of 250 nM PACAP-27 with chelerythrine (20 µM), BIM-1 (500 nM) and Rp-cAMPS (500 µM) in the patch pipette. The lower panel shows the traces in the absence (black) and presence (grey) of PACAP-27 superimposed. The dashed line represents the baseline current. (D) Bar chart summarizing the relative inhibition of sI_AHP_ by 250 nM PACAP-27 under control conditions (*n* = 18; white bar; same data as in Fig. 1F, reported here for comparison) and in the presence of Chelerythrine + BIM-1 (*n* = 5; black bar), Rp-cAMPS (*n* = 15; grey bar; same data as in Fig. 3C, reported here for comparison), and Chelerythrine + BIM-1 + Rp-cAMPS (*n* = 10; stripy bar). Chelerythrine and BIM-1 did not prevent the suppression of sI_AHP_ by PACAP-27 and did not affect the partial inhibition of the PACAP-27 effect by Rp-cAMPS. Error bars indicate S.E.M. * indicates statistical significance.

To obtain maximal PKC inhibition, a combination of two specific inhibitors acting at different sites on PKC, 20 µM chelerythrine (Herbert et al., 1990) and 500 nM bisindolylmaleimide I (BIM-1) (Toullec et al., 1991), was applied intracellularly. Inhibition of sI_AHP_ amplitude (89.3% ± 7.7%, *n* = 5) by PACAP-27 (250 nM) was not significantly different compared to recordings performed in the absence of chelerythrine and BIM-1 (Fig. 7A,B, and D; *P* > 0.3, Mann–Whitney test). An inward shift in the holding current of 29.7 ± 9.1 pA (*n* = 5; *P* = 0.03) was observed upon application of PACAP-27 (250 nM) in the presence of the PKC inhibitors. To validate PKC inhibition under our experimental conditions, we activated PKC by phorbol-12,13-dibutyrate (PDBu). Under control conditions, PDBu (500 nM) caused a strong inhibition of the sI_AHP_ amplitude (74.4% ± 3.3%; *n* = 5), which was significantly reduced (34.7% ± 15.4%, *n* = 6, *P* = 0.04, unpaired *t* test) in the presence of chelerythrine (20 µM) and BIM-1 (500 nM). This indicates that PKC was indeed inhibited under these experimental conditions.

**Figure 8:**
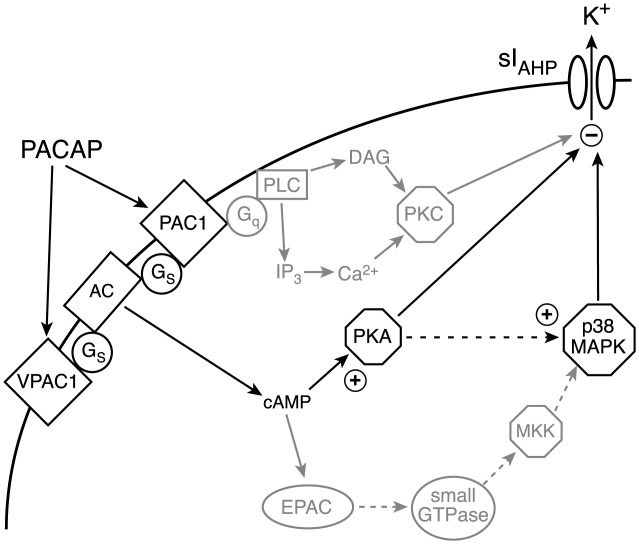
Signal transduction pathway mediating the suppression of sI_AHP_ by PACAP in CA1 pyramidal neurons. Schematic drawing illustrating the signal transduction pathway mediating the effect of PACAP on the sI_AHP_ in CA1 pyramidal neurons. Pathway components supported by the results obtained in our study are depicted in black; other potential contributors, whose role was discounted by the results of our experiments, are shown in grey. AC, adenylyl cyclase; G_s_, G-alpha-s heterotrimeric G-protein; G_q_, G-alpha-q heterotrimeric G-protein; PLC, phospholipase C; DAG, diacylglycerol; IP_3_, inositol 1,4,5 trisphosphate; MKK, mitogen-activated protein kinase kinase.

These experiments show that the PKC signaling cascade does not significantly contribute to the inhibition of the sI_AHP_ by PACAP.

### Is a Crosstalk Between the PKA and PKC Pathways Involved in the Inhibition of sI_AHP_ by PACAP?

The lack of PKC involvement in the suppression of the sI_AHP_ by PACAP inferred from the experiments where only PKC was inhibited does not entirely exclude the possibility of a function of PKC in the context of a crosstalk with the PKA/p38 MAPK pathway. To elucidate this hypothesis we applied PACAP-27 in the presence of both PKA and PKC inhibitors. The inhibition of sI_AHP_ by PACAP-27 was reduced to 56.4% ± 5.0% in the presence of Rp-cAMPS, chelerythrine and BIM-1 (Fig. 7C,D; *n* = 10, *P* = 0.03, Mann–Whitney test). The inhibition of the sI_AHP_ amplitude by PACAP-27 was similar in the presence of Rp-cAMPS alone and the combination of Rp-cAMPS, chelerythrine and BIM-1 (Fig. 7D; *P* > 0.8, Mann–Whitney test). By contrast, inhibition of PKC alone by chelerythrine and BIM-1 with no Rp-cAMPS did not reduce the PACAP effect to a similar extent (Fig. 7D; *P* = 0.003 Mann–Whitney test).

These results suggest that the involvement of PKC in the PACAP-mediated inhibition of the sI_AHP_ is unlikely also as part of a crosstalk.

## DISCUSSION

This study shows that PACAP increases the excitability of CA1 pyramidal neurons by inhibiting the slow Ca^2+^-activated K^+^ current sI_AHP_ through the activation of multiple signaling pathways, most prominently cAMP/PKA and p38 MAPK. Our findings reveal a novel modulation of the sI_AHP_ by p38 MAPK in CA1 pyramidal neurons, adding to the role of this current as a convergency point for modulatory inputs mediated by multiple protein kinases in central neurons.

Previous studies have identified the slow afterhyperpolarization in hippocampal neurons as a target for the neuromodulatory actions of several neuropeptides, including CRF (Aldenhoff et al., 1983; Haug and Storm, 2000), VIP (Haas and Gahwiler, 1992; Haug and Storm, 2000), and CGRP (Haug and Storm, 2000). Similar effects on the sI_AHP_ have been reported for CRF, VIP and PACAP in layer II/III and V neocortical pyramidal neurons (Hu et al., 2011). Our study shows that in CA1 pyramidal neurons both PACAP isoforms, PACAP-38 and PACAP-27, suppress the sI_AHP_, leading to an enhancement of intrinsic excitability and attenuation of spike frequency adaptation. The suppression of the sI_AHP_ by PACAP shown in our study provides a cellular mechanism for the increase in spontaneous firing of CA1 neurons observed *in vivo* upon application of PACAP-38 (Di Mauro et al., 2003).

CA1 pyramidal neurons express all three types of PACAP receptors, PAC_1_, VPAC_1_ and VPAC_2_ (Hashimoto et al., 1996; Shioda et al., 1997; Joo et al., 2004). The ∼30% reduction of the sI_AHP_ amplitude we observed upon application of the selective PAC_1_ agonist maxadilan suggests a potential contribution of PAC_1_ receptors. The presence of the PAC_1_/VPAC_2_ antagonist PACAP-(6–38) (Dickinson et al., 1997; Moro et al., 1999) indeed prevented the effect of maxadilan, supporting a potential role for PAC_1_ receptors. Additionally the activation of VPAC_1_, but not VPAC_2_ receptors, leads to the suppression of the sI_AHP_ amplitude, because the activation of PAC_1_ receptors alone by maxadilan is not sufficient to inhibit the sI_AHP_ to a similar extent as caused by PACAP. The involvement of VPAC_1_ receptors was further inferred by the observation that the PAC_1_/VPAC_2_ antagonist PACAP-(6–38) did not prevent the inhibition of the sI_AHP_ by PACAP. This is different from what has been observed in the neocortex, where PACAP has been reported to activate the cAMP/PKA pathway by acting mainly through PAC_1_ receptors (Hu et al., 2011). Our study concludes that the sI_AHP_ suppression by PACAP can be elicited through the activation of PAC_1_ and most likely VPAC_1_ receptors in CA1 pyramidal neurons.

PAC_1_ and VPAC_1_ receptors can couple to multiple signal transduction pathways, often depending on the cell type and developmental stage. Both receptors activate adenylyl cyclases, leading to the production of cAMP in a variety of cells. Additionally, they can activate phospholipase C, leading to the formation of diacylglycerol and inositol trisphosphate, and phospholipase D (Fig. 8, reviewed in Vaudry et al., 2009). Because the closely related neuropeptide VIP suppresses the sI_AHP_ by activating the cAMP/PKA pathway in CA1 pyramidal neurons (Haug and Storm, 2000) and PACAP has been reported to do the same in the neocortex (Hu et al., 2011), we initially expected PACAP to exert its inhibitory action on the sI_AHP_ predominantly through the same pathway. Surprisingly, inhibition of PKA by the phosphodiesterase-resistant cAMP analogue Rp-cAMPS (Botelho et al., 1988) only partially prevented the effect of PACAP on the sI_AHP_. Interestingly, a similar partial inhibition by Rp-cAMPS was reported for the effect of VIP on the sI_AHP_ (Haug and Storm, 2000). The residual sI_AHP_ inhibition might be a consequence of Rp-cAMPS being a competitive PKA inhibitor that can be partly displaced by high levels of cAMP (Botelho et al., 1988). This would mean that high levels of cAMP, such as those produced by higher concentrations of PACAP, would weaken the inhibitory effect of Rp-cAMPS on PKA. However, our observation that Rp-cAMPS reduced the sI_AHP_ inhibition by higher concentrations of PACAP if anything more than by low concentrations makes this an unlikely explanation. Given the coupling of PACAP receptors to multiple signaling pathways and the modulation of sI_AHP_ by various protein kinases, including PKA, PKC, and calcium-calmodulin protein kinase II (reviewed in Stocker et al., 2004), it is plausible that PACAP suppresses this current by activating also other pathways beside cAMP/PKA. In neurons PACAP activates two different MAP kinases, ERK1/2 and p38 (Sakai et al., 2001; Shi et al., 2006; Monaghan et al., 2008; Stetler et al., 2010). Our data support a significant contribution of p38 MAPK to the PACAP effect on the sI_AHP_. Interestingly, the contribution of p38 MAPK is more prominent than that of PKA for the low concentration of PACAP, while PKA seems to prevail at the high PACAP concentration. The different impact of p38 MAPK in response to different PACAP concentrations might reflect the recruitment of different signaling pathways by signals of different intensities (Dumaz and Marais, 2005). Conversely, inhibition of MEK/ERK1/2 did not affect the inhibition of the sI_AHP_ by PACAP-27 at any concentration tested. On the basis of our data, we propose a novel role for p38-MAPK in the modulation of the sI_AHP_ and intrinsic excitability of CA1 pyramidal neurons.

If PACAP activated the PKA and p38 MAPK pathways in parallel, their concomitant inhibition should lead to an additive reduction of the sI_AHP_ suppression. The lack of additive effect of PKA and p38 MAPK inhibitors in our study suggests that these two kinases act as part of the same signaling pathway, rather than being engaged by two separate pathways activated in parallel by PACAP and converging on the sAHP channels (Fig. 8). Evidence for a sequential action of PKA and p38 MAPK within the same pathway has been found in various cell types, where PKA has been proposed to activate p38 MAPK through an as yet unknown mechanism (Zheng et al., 2000; Delghandi et al., 2005; Shi et al., 2006). A similar signaling pathway might indeed underlie the effect of PACAP on the sI_AHP_ that we have observed in CA1 pyramidal neurons. In view of the results obtained with PACAP in this study, it will be interesting to see whether p38 MAPK might contribute to the neuromodulatory effects also of other neuropeptides and neurotransmitters. Indeed, p38 MAPK has been shown to mediate the persistent sAHP suppression caused by prolonged stimulation of type 5 metabotropic glutamate receptors in CA3 pyramidal neurons (Young et al., 2008).

In other studies, p38 MAPK is a downstream substrate of the cAMP signaling pathway independent of PKA, through the activation of exchange proteins directly activated by cAMP (EPACs) and small G-proteins (Fig. 8, Shi et al., 2006; Ster et al., 2007). Our results, obtained by using a highly specific EPAC superactivator (Rehmann et al., 2003), do not support an involvement of EPACs in the modulation of sI_AHP_.

PAC_1_ and VPAC_1_ receptors are coupled to the phospholipase C (PLC) pathway, leading to the activation of PKC (Fig. 8, reviewed in Vaudry et al., 2009). PKC suppresses the sI_AHP_ in hippocampal pyramidal neurons (Malenka et al., 1986). However, our data show that PACAP does not suppress sI_AHP_ by activating PKC in CA1 pyramidal neurons. This argues in favor of selective coupling between PACAP receptors and specific signal transduction pathways in hippocampal neurons. The basis for this selectivity might lie in the existence of signaling microdomains defining the spatio-temporal dynamics of PACAP signaling, a topic that will be addressed in future studies.

In conclusion, our study shows that PACAP effectively modulates the intrinsic excitability and firing behavior of CA1 pyramidal neurons through the suppression of sI_AHP_. It is well established that a reduction in the sAHP in CA1 and CA3 neurons accompanies acquisition of hippocampus-dependent learning, whereas increases in the sAHP are correlated with cognitive impairment (reviewed in Disterhoft et al., 2004). The impact of the sI_AHP_ on learning processes reflects its multiple roles in controlling firing and intrinsic excitability (Zhang and Linden, 2003) and integration of synaptic signals (Sah and Bekkers, 1996; Borde et al., 1999; Lancaster et al., 2001; Wu et al., 2004; Fernandez de Sevilla et al., 2007). *Drosophila* harboring a mutation in the PACAP-related gene *amnesiac* display deficits in associative learning (Quinn et al., 1979; Feany and Quinn, 1995). In the rat, PACAP intracerebroventricular administration enhances learning, possibly by affecting memory consolidation (Sacchetti et al., 2001). Mice lacking PAC_1_ receptors display impaired long-term potentiation (LTP) of synaptic transmission at several synapses in the hippocampal formation (Otto et al., 2001; Matsuyama et al., 2003), correlating with a deficit in contextual fear conditioning, a hippocampus-dependent learning task (Sauvage et al., 2000; Otto et al., 2001; Matsuyama et al., 2003). PACAP regulates bidirectional synaptic plasticity at the Schaffer collateral CA1 synapse in a concentration-dependent manner, whereby sub-nanomolar concentrations of the peptide induce long-lasting facilitation of excitatory synaptic potentials (Roberto et al., 2001), while concentrations similar to those used in this study (250–500 nM) cause either an initial decrease followed by an enhancement (Roberto et al., 2001), or a long-lasting depression of excitatory synaptic transmission (Kondo et al., 1997; Ster et al., 2009) that depends on EPAC and p38 MAPK activation (Ster et al., 2009). Taken together, our results suggest that modulation of the sI_AHP_ via PAC_1_ and VPAC_1_ receptors might contribute to the complex effects of PACAP on synaptic plasticity in the hippocampal formation and ultimately on learning and memory consolidation.
